# Supplementary material for: Are the Eatwell Guide and Nutrient Profiling Models Consistent in the UK?
Source: Nutrients. 2021 Aug 9;13(8):2732. doi: 10.3390/nu13082732 (PMC8401171; doi:10.3390/nu13082732)
Supplement: Supplementary file 1 [file nutrients-13-02732-s001.zip › nutrients-1309968-supplementary.pdf]

Are the Eatwell Guide and Nutrient Profiling Models consistent in the UK?

Supplementary data

Tables

Table S1: EWG recommendations

**Nutrients**	
**Energy[1]**	2250 kcal (9414 MJ)
**Carbohydrates**	≥50% of food energy
**Free sugars**	≤5% food energy
**Fat**	≤35% food energy
**Saturated fat**	≤11% food energy
**Protein**	≥15.5% of energy
**Salt**	≤ 6g/d (2363 mg sodium/d)
**Fibre (AOAC)**	≥30g/d
**Foods**	
**Fruits and vegetables[2]**	≥5 portions a day
**Fish**	≥ 2 portions (2*140g) a week, one of which should be oily
**Red and processed meat**	≤70g/day

[1] Energy intake recommendation assumes mixed population average, so it was not included in the scoring system

[2] Fruit and vegetable intake includes a maximum of 1 portion of juice (from fruit / vegetable juice or that in a smoothie); 1 portion of beans; (portion sizes: 30 g dried fruit; combined total of 150 ml of fruit and / or vegetable juice and / or smoothie; 80 g all other fruits & vegetables).

Table S2: Basis of the nutrient/food component and scoring for the UK NPM 2005 and the draft 2018 NPM

		**UK NPM 2005**								**Draft 2018 NPM**							
		UK NPM 2004/5 Based on 8,950kJ (2,130kcal) diet, 3.75% scoring bands								Modifications: Based on 8,400kJ (2,000kcal); 3.75% scoring bands							
		'A' points				'C' points				'A' points				'C' points			
		Energy (kJ)	Saturated fat (g)	Total sugars (g)	Sodium (mg)	Protein (g)	FNV (%)	Fibre (AOAC) (g)	Fibre (NSP) (g)	Energy (kJ)	Saturated fat (g)	Free sugars (g)	Salt (g)	Protein (g)	FNV (%)	Fibre (AOAC) (g)	Fibre (NSP) (g)
**Reference**		8,950 kJ	11% of food energy	21% of food energy	2,400 mg	42 g	400 g	24 g	18 g	8,400 kJ	11% of food energy	5% of total dietary energy	6 g	42 g	400 g	30 g	22.5 g
**0**	≤	335	1	4.5	90	1.6	40	0.9	0.7	315	0.9	0.9	0.2	1.6	40	0.7	0.6
**1**	>	335	1	4.5	90	1.6	40	0.9	0.7	315	0.9	0.9	0.2	1.6	40	0.7	0.6
**2**	>	670	2	9	180	3.2	60	1.9	1.4	630	1.9	1.9	0.5	3.2	60	1.4	1.1
**3**	>	1005	3	13.5	270	4.8	-	2.8	2.1	945	2.8	2.8	0.7	4.8	-	2.2	1.7
**4**	>	1340	4	18	360	6.4	-	3.7	2.8	1260	3.7	3.7	0.9	6.4	-	2.9	2.2
**5**	>	1675	5	22.5	450	8	80	4.7	3.5	1575	4.7	4.6	1.1	8	80	3.6	2.8
**6**	>	2010	6	27	540					1890	5.6	5.6	1.4			4.3	3.4
**7**	>	2345	7	31	630					2205	6.6	6.5	1.6			5.0	3.9
**8**	>	2680	8	36	720					2520	7.5	7.4	1.8			5.8	4.5
**9**	>	3015	9	40	810					2835	8.4	8.3	2.0				
**10**	>	3350	10	45	900					3150	9.4	9.3	2.3				

Calculating a nutrient profiling score:

- A maximum of 10 points can be awarded for each nutrient/component (energy, saturated fat, sugar and salt). The total ‘A’ points are the sum of the points scored for each nutrient/ food component. Total 'A' points = [points for energy] + [points for saturated fat] + [points for free sugars] + [points for salt]
- A maximum of 5 points can be awarded for protein and fruit, vegetables and nuts. A maximum of 8 points can be awarded for fibre (either as AOAC or NSP). The total ‘C’ points are the sum of the points for each nutrient/ food component. Total 'C' points = [points for fruit, vegetables and nut content] + [points for fibre (either NSP or AOAC)] + [points for protein]
- The nutrient profiling score is calculated as total ‘A’ minus total ‘C’ points
- If a food or drink scores 11 or more 'A' points then it cannot score points for protein unless it also scores 5 points for fruit, vegetables and nuts.
- A food is classified as 'less healthy' where it scores 4 points or more
- A drink is classified as 'less healthy' where it scores 1 point or more

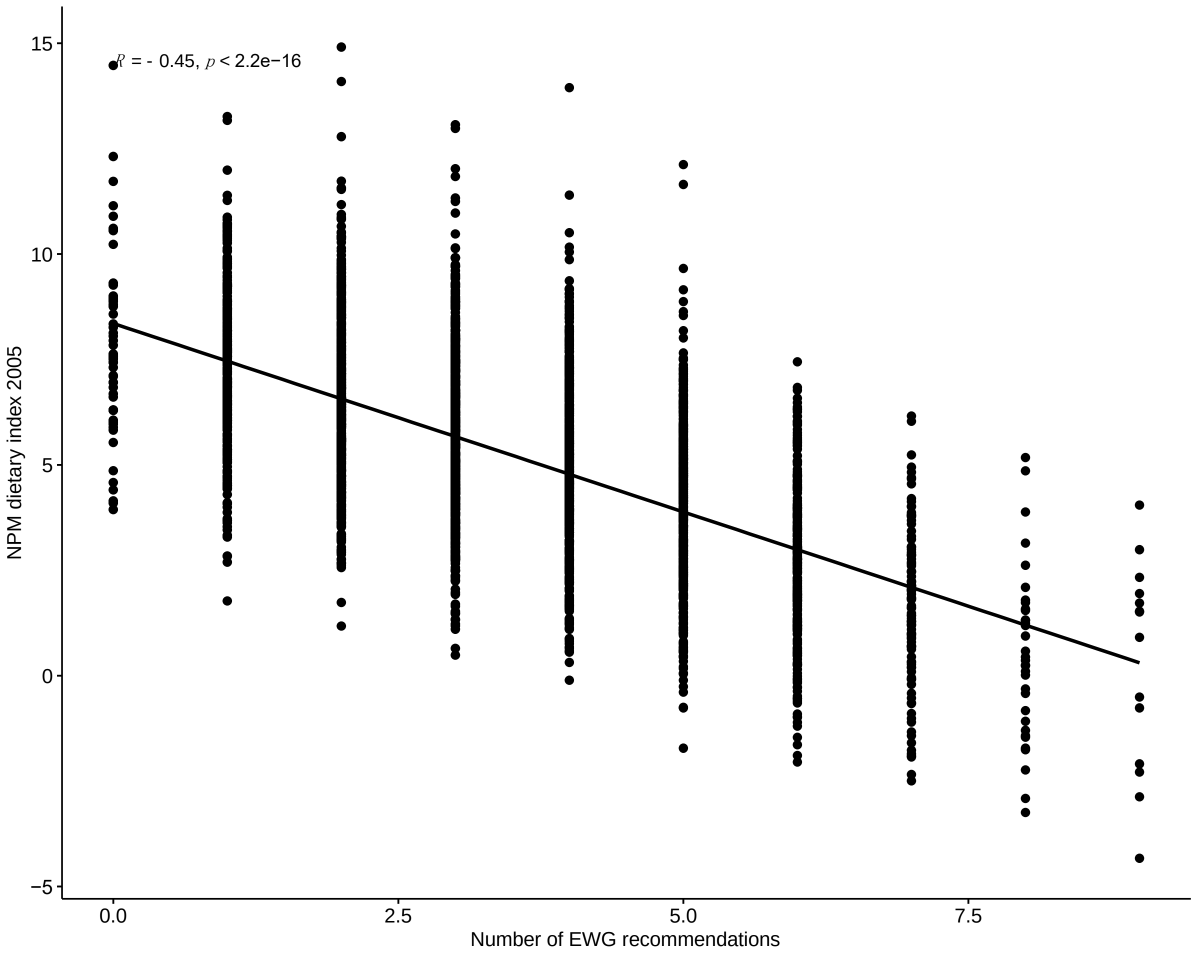

Figure S1: Scatter plot of NPM dietary index 2005 by number of achieved EWG recommendations

The higher the NPM dietary index 2005, the less healthy the diet is; the higher the EWG score, the healthier the diet is.

EWG, Eatwell guide; NPM, nutrient profiling model

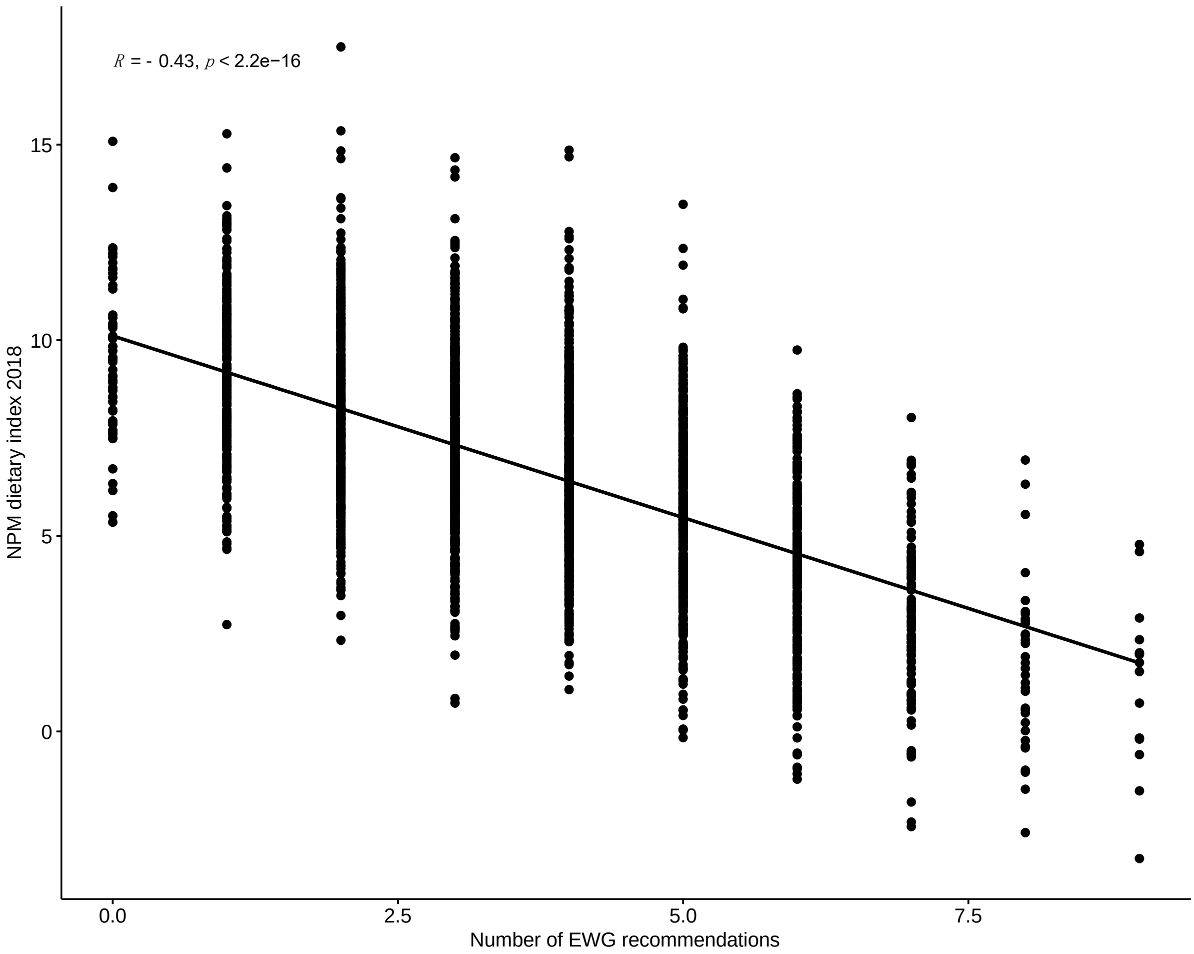

Figure S2: Scatter plot of NPM dietary index 2018 by number of achieved EWG recommendations

The higher the NPM dietary index 2018, the less healthy the diet is; the higher the EWG score, the healthier the diet is.

EWG, Eatwell guide; NPM, nutrient profiling model
